# Observation of Restored Topological Surface States in Magnetically Doped Topological Insulator

**DOI:** 10.1038/s41598-018-37663-8

**Published:** 2019-02-04

**Authors:** Jinsu Kim, Eun-Ha Shin, Manoj K. Sharma, Kyuwook Ihm, Otgonbayar Dugerjav, Chanyong Hwang, Hwangho Lee, Kyung-Tae Ko, Jae-Hoon Park, Miyoung Kim, Hanchul Kim, Myung-Hwa Jung

**Affiliations:** 1 Department of Physics, Sogang University, Seoul, 04107 Korea; 2 Department of Physics, Sookmyung Women’s University, Seoul, 04310 Korea; 3 Department of Applied Physics, Amity Institute of Applied Sciences, Amity University, Noida, 201303 India; 4 Pohang Accelerator Laboratory, Pohang, 37673 Korea; 5 Center for Nanometrology, Korea Research Institute of Standards and Science, Daejeon, 34113 Korea; 6 Department of Physics, Pohang University of Science and Technology, Pohang, 37673 Korea; 7 Max Planck POSTECH Center for Complex Phase Materials, Pohang University of Science and Technology, Pohang, 37673 Korea; 8 Division of Advanced Materials Science, Pohang University of Science and Technology, Pohang, 37673 Korea

## Abstract

The introduction of ferromagnetic order in topological insulators in general breaks the time-reversal symmetry and a gap is opened in topological surface bands. Various studies have focused on gap-opened magnetic topological insulators, because such modified band structures provide a promising platform for observing exotic quantum physics. However, the role of antiferromagnetic order in topological insulators is still controversial. In this report, we demonstrate that it is possible to restore the topological surface states by effectively reducing the antiferromagnetic ordering in Gd-substituted Bi_2_Te_3_. We successfully control the magnetic impurities via thermal treatments in ultra-high vacuum condition and observe apparent restoration of topological surface band dispersions. The microscopic mechanism of atomic rearrangements and the restoration process of topological surface states are unraveled by the combination of scanning tunneling microscopy measurements and density functional theory calculations. This work provides an effective way to control the magnetic impurities which is strongly correlated with topological surface states.

## Introduction

Bismuth chalcogenides have attracted great attention in condensed matter physics as well as materials science because they hold enormous potential as thermoelectric materials^1,2^ and topological insulators (TIs)^3–7^. Especially, TIs offer a new platform for the study of topological properties such as quantum linear magnetoresistance^8^, spin-polarized two-dimensional transport^3^, and Majorana fermions^9^. The most prominent property of TIs lies in gapless surface states, which is topologically protected by time-reversal symmetry (TRS)^10,11^. Various research groups have reported the effect of impurities on the gapless surface states with linear energy dispersion^12–16^. Nonmagnetic impurities such as NO_2_, Ca, and Rb give almost no effect on the linear energy dispersion but play an important role in tuning the Fermi level^12,13^, preserving the topological surface states (TSS). The electrical transport of spin-momentum-locked TSS is believed to be robust in the presence of nonmagnetic perturbations. However, TRS can be broken under magnetic perturbations within the proximity to magnetic materials. Local ferromagnetic phases or magnetic impurities such as Cr, Mn, and Fe induce an energy gap at the Dirac point^4,7,11,17^, which modifies the energy dispersion relation of TSS. Most of previous works focus on the gap opening of surface band by doping magnetic impurities into TIs. One meaningful question is whether the TSS nature once destroyed by the magnetic impurities can be restored by controlling such magnetic perturbation.

To solve such curiosity, we choose Gd substituted TIs, because the trivalent nature of Gd ions tunes only the magnetism in TIs. In the previous report on Gd-substituted Bi_2_Te_3_, Gd_*x*_Bi_2−*x*_Te_3,_ non-trivial topological features were observed near the magnetic phase transition at *x*_c_ = 0.09 from paramagnetic (PM) to antiferromagnetic (AFM) phase^18^. The experimental observations near *x*_c_ are high electrical resistivity, large linear magnetoresistance, and highly anisotropic Shubnikov-de Haas (SdH) oscillations with two-dimensional (2D) TSS origin. Interestingly, the samples of *x* > *x*_c_ show AFM ordering and normal metallic behavior without 2D Dirac fermion dynamics. It is not clear that such topologically trivial phase could be caused by the AFM evolution in Gd_*x*_Bi_2−*x*_Te_3_. In theoretical expectation, AFM TI state is achieved for a particular combination of both TRS and translation symmetry breaking, and one of AFM TIs candidates is half-Heusler antiferromagnet GdBiPt^19^. On the other hand, there are some experimental observations of competing features between AFM order and topologically non-trivial phase^20,21^. To elucidate such ambiguity for the role of antiferromagnetism in TIs, the diverse reports on the TSS characteristics are needed as the AFM ordering evolves.

In this study, we investigate various physical properties of antiferromagnetically ordered Gd_0.15_Bi_1.85_Te_3_ single crystals while focusing on the TSS properties affected by magnetic ordering. We have successfully controlled the magnetic impurity populations by thermal treatments in ultra-high vacuum (UHV) condition, and have observed the improvement of 2D TSS characteristics such as large linear magnetoresistance, SdH oscillations with 1/2 offset, and multiband Hall behavior. These results are clearly explained by the restoration of TSS band dispersion, which is detected in angle-resolved photoemission spectroscopy (ARPES) measurements. The detailed dynamics of the TSS restoration are discussed with the combination of scanning tunneling microscopy (STM) measurements and density functional theory (DFT) calculations. By engineering the magnetic impurity sites via thermal treatments in UHV condition, we achieve the effective reduction of AFM ordering, leading to the restored topological surface states in magnetically doped TIs. These observations of the magnetic correlation with TSS provide a meaningful information in the field of magnetic TIs and topological antiferromagnetic spintronics^22^.

## Results and Discussion

### Atomic migration after vacuum annealing

**Figure 1:**
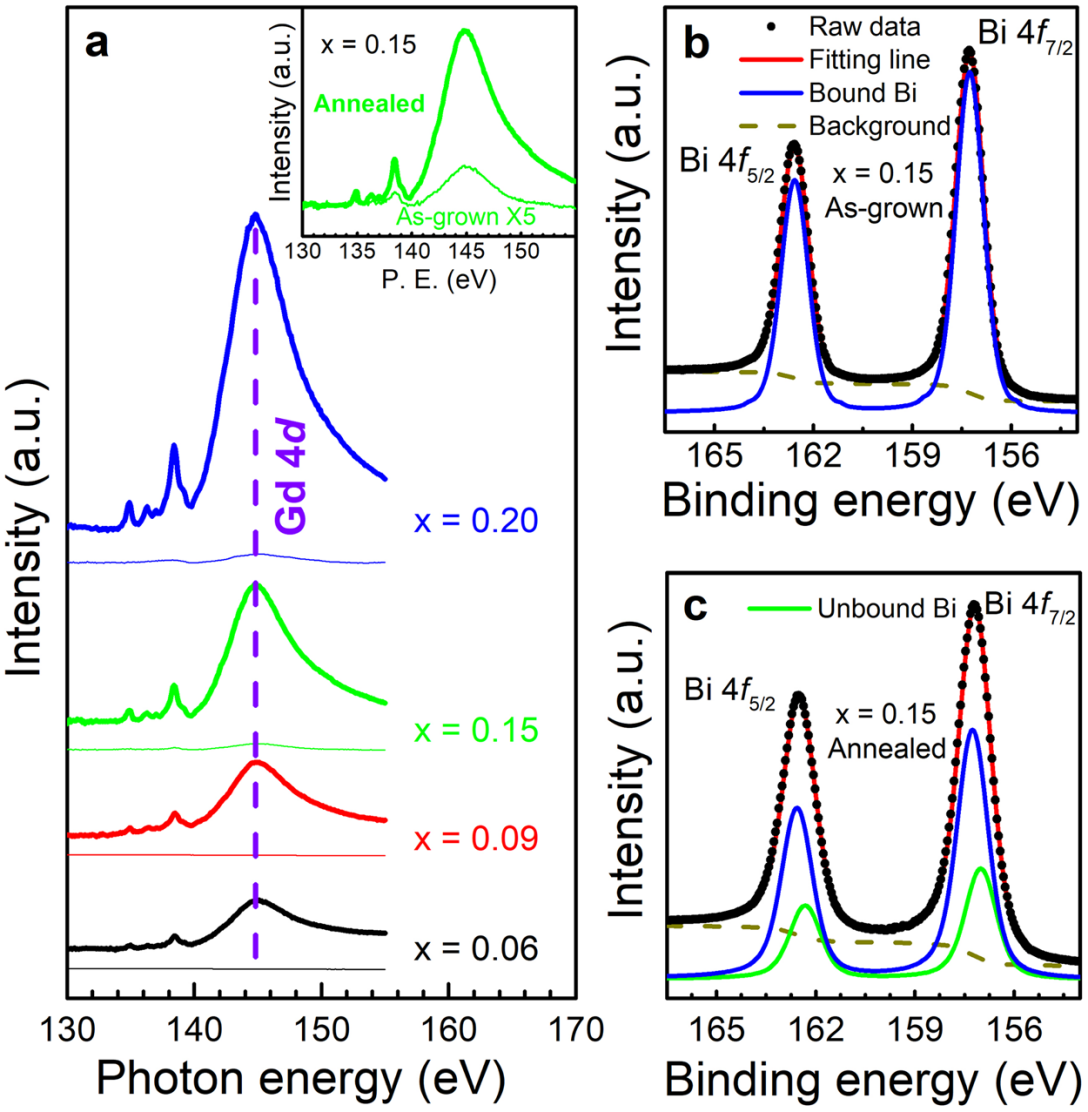
Near edge x-ray absorption fine structure (NEXAFS) and x-ray photoelectron spectroscopy (XPS) of Gd_*x*_Bi_2−*x*_Te_3_. (**a**) Gd N-edge NEXAFS spectra taken for as-grown and annealed samples with various *x* (=0.06, 0.09, 0.15, and 0.20). The thin and thick lines correspond to the spectra for as-grown and annealed samples, respectively. The inset shows a typical comparison of the NEXAFS spectrum at *x* = 0.15 before and after annealing. The data of as-grown sample have been multiplied by 5. (**b**,**c**) Bi 4 *f* core-level XPS spectra of *x* = 0.15 taken for as-grown and annealed samples, respectively. Before annealing, the data are fitted using two peaks for Bi 4*f*_7/2_ and Bi 4*f*_5/2_ with binding energies of 157.0 and 162.3 eV, in addition to a normal-state background signal (dashed line). After annealing, the data are resolved into two chemical states of bound Bi (blue line) and unbound Bi (green line) with a slight energy shift, about 0.2 eV.

We have first examined the chemical state of elements of Gd_*x*_Bi_2−*x*_Te_3_ samples by near edge x-ray absorption fine structure (NEXAFS) and x-ray photoelectron spectroscopy (XPS) measurements, which especially give information about chemical bonding state on the surface. The spectra were taken for as-grown samples (called “as-grown”) and *in-situ* annealed samples in an ultra-high vacuum (called “annealed”). Figure 1a shows Gd N-edge NEXAFS spectra of Gd_*x*_Bi_2−*x*_Te_3_ (*x* = 0.06, 0.09, 0.15, and 0.20) samples. The sharp peak at 144.8 eV is assigned to the 4*d*–4*f* transition of Gd^23^. The spectral intensity of this Gd peak increases with increasing the Gd concentration, *x*, and it is enormously enhanced for all the samples after annealing. In the inset of Fig. 1a, a typical intensity enhancement after annealing is clearly shown in a magnified scale. This observation implies that Gd atoms are more populated at the surface after the vacuum annealing, possibly due to migration from the bulk inside. Similarly, the chemical state of Bi element is determined from the XPS spectra. Figure 1b,c display the Bi 4 *f* core-level XPS spectra for *x* = 0.15 before and after annealing. The Bi 4 *f* spectra show a simple spin-orbit doublet structure. The two main peaks around 157.0 and 162.3 eV correspond to the binding energy of Bi 4 *f*_7/2_ and Bi 4*f*_5/2_, respectively. Each core-level spectrum was fitted with two components using a least-squares fitting and mixed Gaussian-Lorentzian functions. An additional asymmetry parameter was used for metallic components in higher binding energy side^24,25^. In Fig. 1b, the Bi 4*f* spectrum for as-grown sample is fully fitted only with bound Bi states after subtraction of a normal state background, in good agreement with the spectra obtained from pristine Bi_2_Te_3_. After annealing in Fig. 1c, the spectrum is resolved into two chemical states of bound Bi and unbound Bi. The unbound Bi peaks have a slight shift (~0.2 eV) towards the lower binding side from the bound Bi peaks. The area ratio of the bound Bi is relatively reduced with the increased portion of unbound Bi, indicating that the Bi ion breaks the bonds with the Te ions and resides at the surface as metallic Bi states. On the other hand, the XPS spectra of Te 3*d* core level did not show any significant difference after annealing (see Supplementary Fig. S3). From the combined NEXAFS and XPS investigations, we speculate that the Gd ions migrate from the bulk inside to the surface during the vacuum annealing, and that the surface Bi atoms attain increased metallic nature.

### Changes of physical properties after vacuum annealing

**Figure 2:**
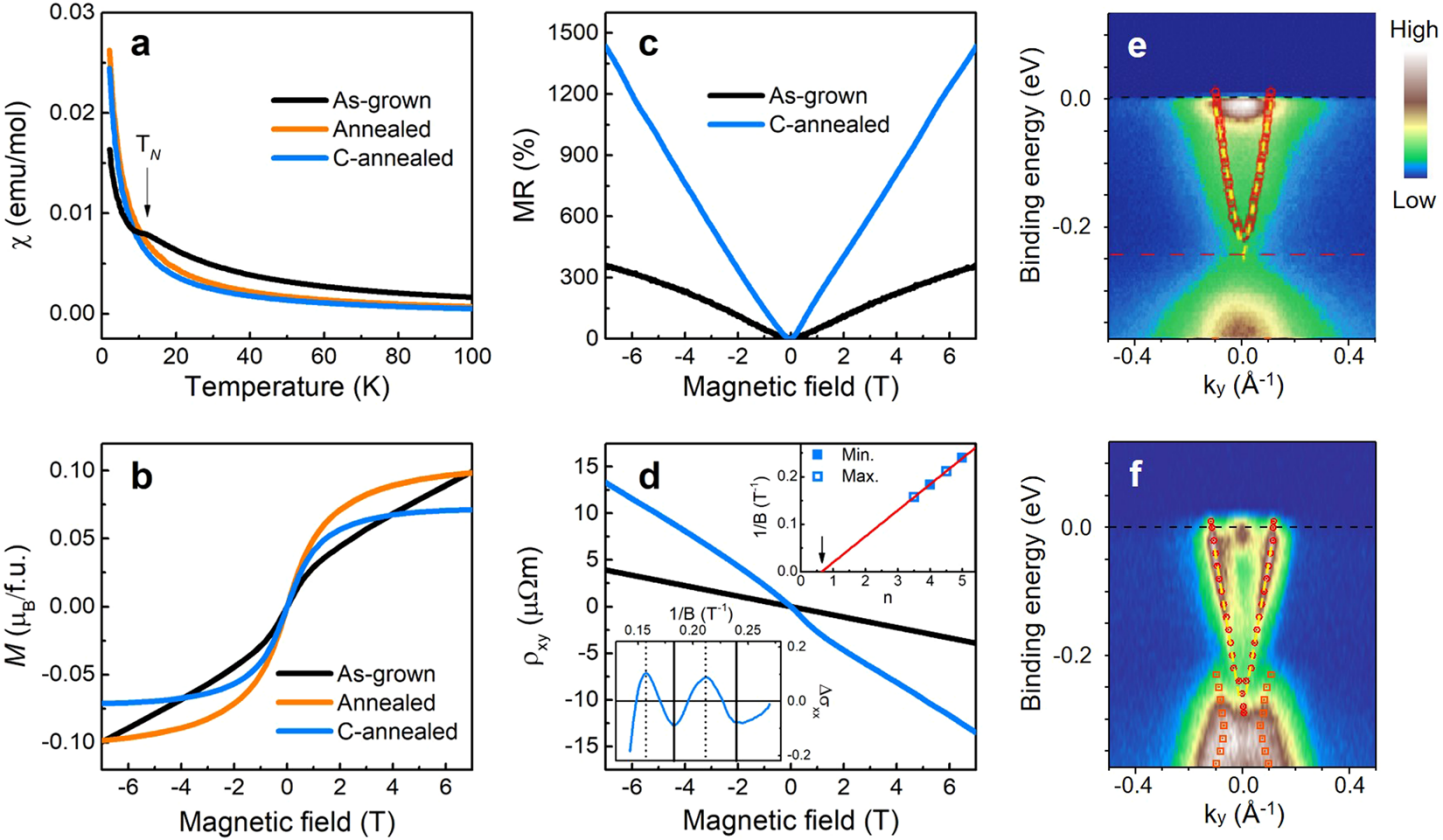
Changes in physical properties and band structure upon annealing. (**a**) Magnetic susceptibility as a function of temperature, *χ*(*T*) measured at 1 T. For the as-grown sample, the arrow represents the Néel temperature *T*_N_ = 11.5 K. Both annealed and c-annealed samples show no such a feature of antiferromagnetic transition. (**b**) Magnetization as a function of the applied magnetic field, *M*(*H*) measured at 2 K. A diverging signal for the as-grown sample changes into saturating behavior for both annealed and c-annealed samples. (**c**) Magnetoresistance, MR ratio measured at 2 K. The c-annealed sample shows large and linear MR behavior. (**d**) Hall resistivity, *ρ*_*xy*_ measured at 2 K. The c-annealed sample shows a bent curve at low fields (blue line), distinguished from the linear behavior of the as-grown sample (black line). The lower inset represents the quantum oscillations of electrical conductivity Δ*σ*_*xx*_, from which the Landau level fan diagram is plotted in the upper inset. The arrow indicates the phase offset, close to 1/2 expected for the Dirac fermions. (**e**,**f**) Angle-resolved photoemission spectroscopy (ARPES) spectra of *x* = 0.15 taken for as-grown and c-annealed single crystals, respectively, measured at 20 K. The as-grown sample shows an energy gap of 60 meV at the Dirac point, while after annealing the energy gap is closed and the surface states are restored.

We have next examined the effect of annealing on the magnetic properties such as the magnetic susceptibility *χ*, and the magnetization *M*. According to previous studies on Gd_*x*_Bi_2−*x*_Te_3_ in ref.^18^, there is a magnetic transition from PM to AFM at the critical Gd composition of *x*_c_ = 0.09. Among them, we chose an AFM crystal of *x* = 0.15, because the Gd atoms are more probable to relocate by annealing in Gd-richer samples and thereby the AFM phase can be changed. As seen in Fig. 2a, the *χ*(*T*) curve of as-grown sample shows an AFM feature with the Néel temperature, *T*_N_ = 11.5 K, which well agrees with the previous data^18^. Interestingly, this AFM fingerprint disappears in two annealed samples; one is the annealed sample and the other is the sample left after the removal of the surface part by cleaving the annealed sample (called “c-annealed”). There is no significant difference of *χ*(*T*) behavior between annealed and c-annealed samples except the magnitude. The magnitude for the c-annealed sample is lower than that for the annealed sample. Reminding that more Gd atoms are present on the surface after annealing, this result is due to the reduction of magnetic moments as a result of removal of the Gd-rich surface part. Since the magnetic measurement is a bulk property, we can evaluate the physical parameters with the bulk origin. From the Curie-Weiss fit in annealed samples, we obtain the Weiss temperature of *θ*_p_ = −0.67 K, which is almost identical value (*θ*_p_ = −0.38 K) for the critical composition of *x*_c_ = 0.09 in ref.^18^. Another fitted parameter of effective magnetic moment gives an important information on Gd concentration.

The c-annealed sample shows the magnetic moment similar to that of the as-grown sample with *x* = 0.10 (~*x*_c_). In a similar way, the *M*(*H*) data can be understood. The *M*(*H*) curve in Fig. 2b tends to increase before annealing, while it tends to saturate after annealing. The previous *M*(*H*) data revealed the diverging signal for AFM (*x* > *x*_c_) and the saturating behavior for PM (*x* ≤ *x*_c_)^18^. The sample with *x* = 0.15 (>*x*_c_) seems to turn into a sample with *x* = 0.10 (~*x*_c_) after annealing. Thus, we expect the non-trivial topological features after annealing, as observed at *x*_c_ in the previous work^18^, so that we check this scenario by measuring the magneto-transport properties such as the magnetoresistance (MR) ratio and Hall resistivity *ρ*_*xy*_.

The MR ratio as a function of applied magnetic field is plotted in Fig. 2c. The MR ratio value at 7 T is 360% for as-grown sample, and after annealing it enormously increases up to around 1400%, close to the MR value for the critical composition *x*_c_. Such large and linear MR behavior is considered as a signature of the 2D properties of TSS electrons. This tendency well agrees with the magnetic data shown in Fig. 2a,b. The Gd concentration is reduced from *x* = 0.15 (that is AFM mainly having bulk properties) to *x* = 0.10 (that is PM exhibiting 2D properties of TSS) by annealing. Another evidence on the existence of TSS after annealing can be found in the Hall measurements. In Fig. 2d, the Hall curve measured with the c-annealed sample shows a weak modulated signal at low fields, so-called bent Hall effect, which occurs via two parallel conducting channels of bulk and surface carriers^26–29^. This result is in contrast with the linear behavior of *ρ*_*xy*_ in the as-grown sample. Also, the coexistence of surface and bulk carriers may construct the charge inhomogeneous system, and these play an importance role in the large linear MR^29^.

Further evidence of the TSS electrons can be found in quantum oscillations. This measurement gives critical insight in the information of Fermi surface. For the c-annealed sample, the MR curve in Fig. 2c shows SdH oscillations at high magnetic fields. Since the resistivity tensor is an inverse of the conductivity tensor, we took the conductivity, *σ*_*xx*_ = *ρ*_*xx*_/(*ρ*_*xx*_^2^ + *ρ*_*xy*_^2^) with respect to magnetic field and plotted it against the inverse of magnetic field. After subtracting background signals with polynomial form, the lower inset of Fig. 2d clearly shows SdH oscillation above 0.15 T^−1^. By identifying the minima to integer *n* and the maxima to *n* + 1/2, we constructed the Landau level (LL) fan diagram in the upper inset of Fig. 2d. The linear fit of the LL fan diagram extrapolates to 0.62 ± 0.04 on the *n*-index axis, which is close to the value 1/2 expected for the Dirac fermions. The small deviation from 1/2 is reasonable because the bulk electrons also in part contribute to the conduction, as discussed above in the bent Hall curve.

### Restoration of TSS band structure in annealed sample

To directly examine how the TSS is evolved after annealing, we performed the ARPES experiments. In the as-grown sample, we observe a gap opening at the Dirac point with the gap size of 60 meV, as shown in Fig. 2e. Such a gap opening and the AFM nature upon Gd substitution suggest that the band topology and the magnetic phase are possibly interconnected, thereby losing the 2D properties of TSS. Note that the ARPES spectra have been taken at 20 K, which is higher than the Néel temperature. So, we consider one possible origin, which is non-magnetic resonant scattering process suggested by Sánchez-Barriga^16^. According to Sánchez-Barriga, non-magnetic bulk impurities could create the surface band gaps of the order of ~100 meV without magnetic moments. To examine this suggestion, we compare some experimental results in similar systems. For example, Ce_*y*_Bi_2−*y*_Te_3_ and Gd_*z*_Bi_2−*z*_Se_3_ single crystals show antiferromagnetic ordering at *y* ≥ 0.08 and *z* ≥ 0.30, respectively, and their transport results reveal that the topologically non-trivial properties disappear in the antiferromagnetic states^20,21^. In the present work on Gd_*x*_Bi_2−*x*_Te_3_, we observe no gap opening in the paramagnetic states (*x* ≤ 0.10), but gap opening in the antiferromagnetic states (*x* ≥ 0.15). These results indicate a close correlation between the topological phase and the antiferromagnetic ordering, leading to possible origin of antiferromagnetism for the band gap opening in Gd_*x*_Bi_2−*x*_Te_3_. Therefore, we reach the conclusion that the surface band gap opening is affected by the magnetic perturbation, rather than the non-magnetic resonant scattering processes, even though we cannot perfectly exclude the non-magnetic origin. In order to check the strength of antiferromagnetic order, we plot the magnetic susceptibility as a function of temperature and fit the data by using the Curie-Weiss law. As seen in Supplementary Fig. S4, the magnetic susceptibility data tend to deviate from the Curie-Weiss fit below 20 K. This result yields that the antiferromagnetic interaction starts near that temperature, which may affect the band gap opening of the as-grown sample.

After vacuum annealing, intriguingly, the surface energy gap disappears, and the linear energy dispersion of TSS is restored as shown in Fig. 2f. The gap closing feature is more visible in the momentum distribution curves, plotted in Supplementary Fig. S5. This observation is consistent with the results discussed above. The restoration of TSS after annealing closes the energy gap, revealing the non-trivial topological properties such as large linear MR, SdH oscillations with 1/2 offset, and multiband Hall behavior.

### Microscopic understanding of annealing effect by STM measurements and DFT calculations

**Figure 3:**
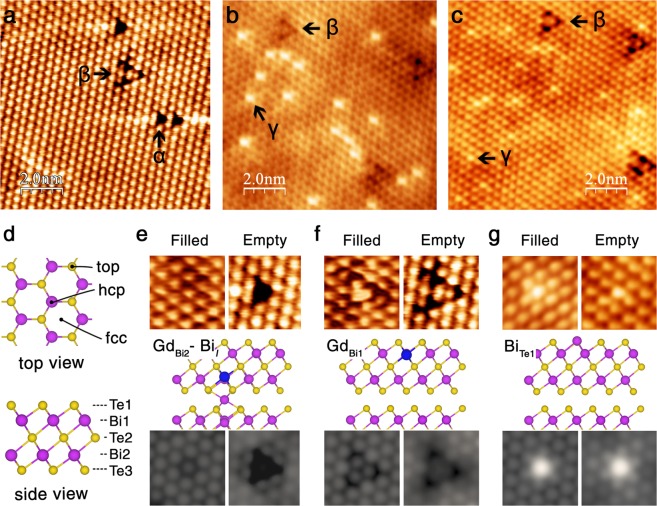
Experimental and simulated STM images of *x* = 0.15 before and after annealing. (**a**) Empty-state scanning tunneling microscopy (STM) image for as-grown single crystal. The defects α and β are related with the Gd substitution. (**b**,**c**) Filled- and empty-state STM images after annealing. Notice that the α defects almost disappear, the β defects increase, and the γ defects are newly formed. (**d**) Schematic diagram of the atomic structure of pristine Bi_2_Te_3_: top view (upper) and side view (lower). The pink and yellow circles represent Bi and Te atoms, respectively. (**e**–**g**) Experimental high-resolution STM images (upper), atomic structures (middle), and simulated constant-current STM images (lower). (**e**) α defect: Gd_Bi2_-Bi_*I*_, (**f**) β defect: Gd_Bi1_, and (**g**) γ defect: Bi_Te1_. The simulated STM images are in good agreement with experiments. The blue circle represents Gd atom. The filled- and empty-state images are measured at *V*_S_ = −0.35 and +0.30 V in as-grown sample and *V*_S_ = −0.65 and +0.35 V in annealed sample, respectively, and simulated at *V*_S_ = −0.6 and +0.6 V, respectively.

To understand the restored mechanism of TSS at the atomic scale, we performed STM measurements and DFT calculations. Figure 3a is a typical empty-state STM images of the (111) cleavage surface of as-grown samples, where Te atoms on the top are imaged as bright protrusions. Native defects, observed in pristine Bi_2_Te_3_ sample, like Te_Bi_-type antisites and Bi vacancies are commonly observed in all Gd-containing samples (A, B, and C defects in Supplementary Fig. S6). Upon Gd substitution, the density of the native defects is significantly reduced and two new Gd-induced defects, α and β, are observed. The defect α appears as a triangular dark depression occupying the top surface Te site, and the defect β appears as a three-leaf-clover-shaped dark depression centered at the hcp site which is on top of the second atomic layer (Bi1). The annealed sample reveals quite different features from the as-grown sample, as shown in Fig. 3b,c. Compared to Fig. 3a, the α defects almost disappear, the β defects increase, and the γ defects are newly formed. The γ defect appears as a bright protrusion at the surface Te site in both filled- and empty-state images.

Possible defects induced by Gd substitution are adsorption on the surface (Gd_ad_), intercalation in the van der Waals gap (Gd_*I*_), and substitution for Bi (Gd_Bi_) or Te (Gd_Te_). From the top view of the crystal, there are three different sites; top surface Te site (top), on-top of Bi1 site (hcp), and the hollow site (fcc), as indicated in Fig. 3d. The atomic layers in a quintuple layer (QL) of Bi_2_Te_3_ are denoted by Te1-Bi1-Te2-Bi2-Te3 from the top surface. Our DFT total energy calculations (see Table S1 of the Supplementary Information) show that the substitutional Gd at Bi sites (Gd_Bi1_ and Gd_Bi2_) are the most stable configurations with the energy difference as small as 5 meV. The next stable structure is a pair of Gd_Bi2_ and Bi_*I*_ (Gd_Bi2_-Bi_*I*_), where Bi_*I*_ is the interstitial Bi in the van der Waals gap, formed via the kick-out of Bi by Gd_Bi2_. The Gd_Bi2_-Bi_*I*_ pair is followed by the interstitial Gd (Gd_*I*_) in the van der Waals gap, but its formation energy is larger than that of Gd_Bi2_-Bi_*I*_ by 0.18 eV. These results are in accordance with the previous reports of preferential substitution of Bi by transition-metal elements such as Cr, Mn, and Fe^30^. Gd adatoms on the surface is unlikely to exist because the formation energy ranging from 4.84–7.30 eV is much larger than those of substitutional Gd defects.

In order to identify the Gd-related defects (α, β, and γ), we simulated STM images for all possible defect types. In the enlarged STM images of Fig. 3e, the α defect appear as a dimmer protrusion taking a single Te site in the filled state and a single Te depression in the empty state. The simulated STM images of the Gd_Bi2_-Bi_*I*_ pair are in good agreement with experiments. As for the β defect, the STM images in Fig. 3f show a triangular bright protrusion in the filled state and a three-leaf-clover-shaped dark depression in the empty state. Such experimental features are well reproduced by the simulation of Gd_Bi1_. Similar STM features have been reported for Fe-doped Bi_2_Te_3_^31^, Mn-doped Bi_2_Te_3_^14^, Cr-doped Sb_2_Te_3_^32^, and Ca-doped Bi_2_Se_3_^33^, and were attributed to the impurity elements substituted for Bi1 sites. In Fig. 3g, the γ defects appear as a bright protrusion on the top Te sites in both filled- and empty-state images, and these defects are identified to be Bi_Te1_ antisite defects based on the good agreement between experimental and simulated images. It is noticeable that the Bi_Te_-type antisite defect is reported to have low formation energy under Bi-rich condition^34–36^, where a clover-shaped protrusion is assigned to the Bi_Te3_ antisite. The relative energetic stability of Bi_Te1_ and Bi_Te3_ in a slab geometry has not been studied yet, and our calculations show that Bi_Te3_ is energetically more favorable than Bi_Te1_ by 0.2 eV. This energetic favor of Bi_Te3_ is in apparent contradiction to the sole observation of Bi_Te1_ in the annealed sample, which suggests that the formation of the γ defect is driven by kinetics rather than by thermodynamics.

**Figure 4:**
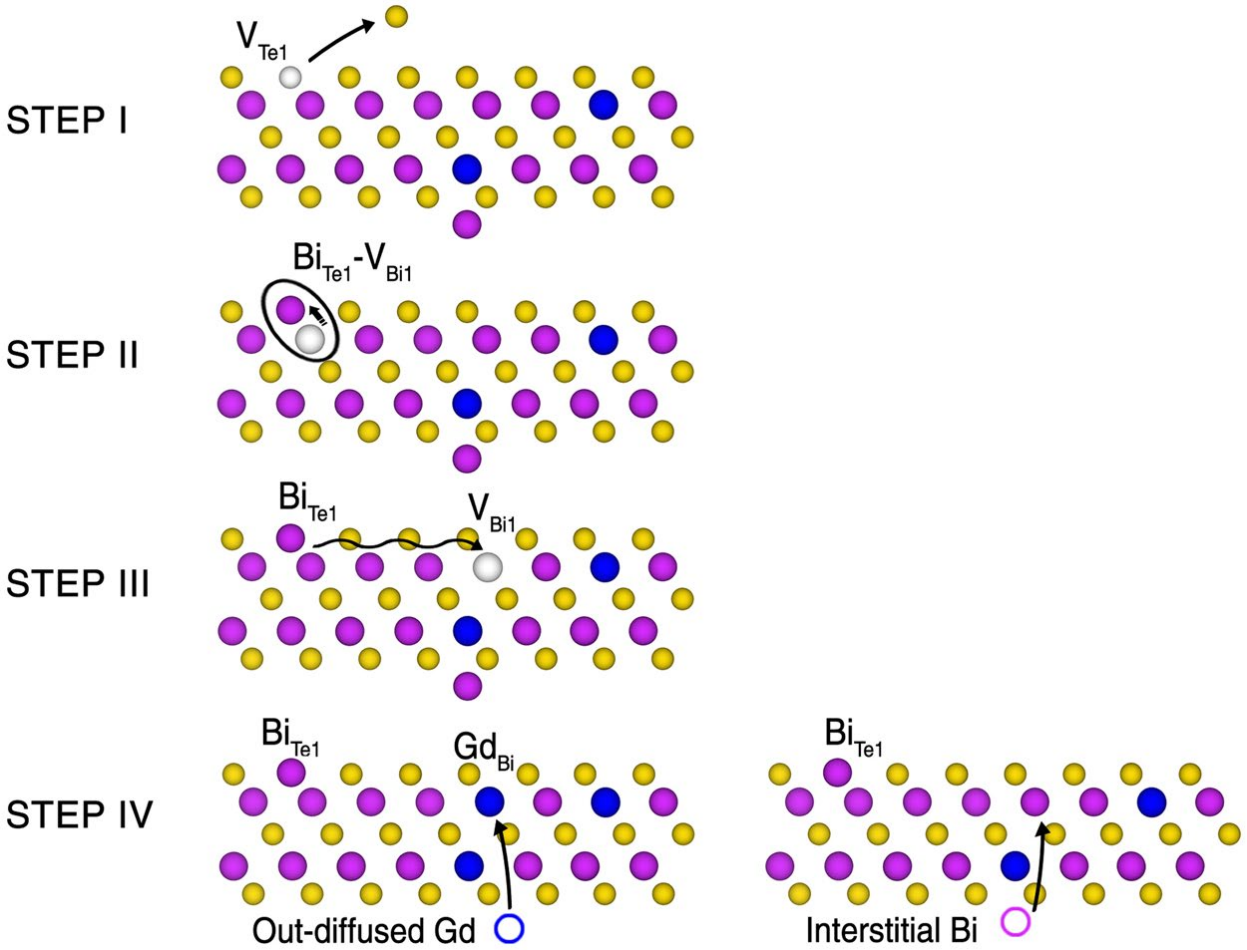
Schematics of a possible atomic rearrangement process during vacuum annealing. In step I, V_Te1_ is formed by the evaporation of surface Te atoms during heating. In step II, a Bi_Te1_-V_Bi1_ pair is formed by the migration of Bi atom from an adjacent Bi site. In step III, V_Bi1_ is liberated to form isolated Bi_Te1_. In step IV, V_Bi1_ is filled either by out-diffused Gd to generate Gd_Bi1_ (β defect; left) or by the interstitial Bi of a Gd_Bi2_-Bi_*I*_ pair to generate Bi_Te1_ (γ defect; right). Notice that the final structure of the right process of step IV, Gd_Bi2_, is indistinguishable from the pristine surface (see Supplementary Fig. S7).

In short, the Gd-related defects, α, β, and γ are identified to be the Gd_Bi2_-Bi_*I*_ pair, the substitutional Gd_Bi1_, and the Bi_Te1_ antisite, respectively. The appearance of α and β defects in the as-grown sample is due to the relatively low formation energy of substitutional Gd_Bi_. After annealing, the disappearance of Gd_Bi2_-Bi_*I*_, the increase of Gd_Bi1_, and the appearance of Bi_Te1_ can be schematically speculated, as depicted in Fig. 4. The volatile Te ions are evaporated upon heating, mostly from the top surface layer, generating V_Te1_ [step 1]. Then, the region near the surface becomes effectively Bi-rich (or Te-poor) phase, compared to the Te-rich condition of the as-grown sample. This change is in commensurate with the disappearance of the native defects (see Supplementary Fig. S6) which are stable in the Te-rich condition. The Te vacancies can be occupied by the adjacent Bi atoms to form the Bi_Te1_-V_Bi_ pair [step 2].

The subsequent liberation of V_Bi_ can result in the creation of Bi_Te1_, the γ defect [step 3]. Then, the liberated V_Bi_ can be occupied by the out-diffused Gd to form a Gd_Bi1_, resulting in the increase of β defect. Alternatively, the liberated V_Bi_ can be occupied by the interstitial Bi in the van der Waals gap, Bi_*I*_ of the Gd_Bi2_-Bi_*I*_ pair. This causes the consumption of the Gd_Bi2_-Bi_*I*_ pairs, removing the α defects. The isolated Gd_Bi2_ defects after consuming the Bi_*I*_ atoms are invisible in the STM images (see Supplementary Fig. S7). Such atomic rearrangements result in a Gd-rich regime near the surface and the relatively dilute Gd bulk phase, which leads to a reduction of magnetic moment and the restoration of TSS.

In addition, the core-level shift calculations within the initial state theory show that the binding energy (BE) of Bi is shifted by −1.1 and −0.3 eV for Bi_Te1_ antisite (γ defect) and its first nearest neighbor Bi atom, respectively. This is in good agreement with the observed BE shift towards lower binding energy side, as shown in Fig. 1c. For Bi_*I*_ in the Gd_Bi2_-Bi_*I*_ pair (α defect), the calculated core-level shift is as small as 0.2 eV towards higher energy side. As for Gd_Bi1_ antisite (β defect), there is no appreciable core-level shift of neighboring Bi atom (0.01 eV towards higher energy side). Our DFT calculations enables the identification of defects formed upon annealing and enlightens the possible atomistic processes.

From the combination of STM measurements and DFT calculations, we find that there are two main atomic rearrangements by vacuum annealing; one is the out-diffusion of Gd to the surface and the other is the formation of unbound Bi on the surface in the form of Bi_Te1_. These results not only agree with our NEXAFS and XPS measurements, but also explain well the magnetic and magneto-transport data. These atomic rearrangements give rise to an effective reduction of magnetic strength, which results in magnetic transition from AFM phase to PM phase. After vacuum annealing, we observe the topologically non-trivial transport properties like large linear MR, 1/2 Berry phase from 2D Dirac fermions, and multiband Hall effect. Finally, we can detect clear restoration of linear band dispersions with 2D TSS nature through ARPES measurements in vacuum annealed sample. These observations demonstrate that there are intimate connections between AFM ordering and TSS characteristics, and such correlation can be effectively controlled by atomic rearrangements. These observations may provide meaningful inspirations to generate stable topological states in magnetically doped TIs and topological AFM spintronics.

## Methods

### Single crystal growth and structural characterization

The single crystals of Gd_*x*_Bi_2−*x*_Te_3_ were synthesized by melting method in a vertical tube furnace with local temperature gradient. A mixture of Bi (99.999%), Te (99.9999%), and Gd (99.99%) with the stoichiometric ratio was put into a cleaned quartz tube with 5% excessive Te and sealed in vacuum. The Te excess acts to compensate the Te loss caused by its high vapor pressure, and the Te-rich condition tends to generate native Te_Bi_-type antisite defects, leading to *n*-type charge carriers. The mixture in the evacuated quartz ampoule was melted at 800 °C and annealed at 550 °C for 3 days. The obtained ingots were well cleaved with a mirror-like surface perpendicular to the c axis. Single-crystal XRD data show (003) family reflections for all samples without any impurity and secondary phases (see Supplementary Fig. S1).

### XPS, NEXAFS, and ARPES measurements

The samples (Gd_*x*_Bi_2−*x*_Te_3_) were loaded and *in-situ* cleaved in an ultra-high vacuum (UHV, base pressure: 1 × 10^−10^ Torr) for XPS, NEXAFS and ARPES measurements. The *in-situ* annealing (250 °C for 2 h), and characterization of the samples by XPS and NEXAFS spectra (4D beamline) and ARPES (4A1 beamline) were performed at Pohang Accelerator Laboratory (PAL). The *in-situ* ARPES measurements were carried out at 20 K by using a Scienta SES2002 electron energy analyzer. XPS and Total Electron Yield (TEY) NEXAFS spectra were recorded at 45° angle of x-ray incidence at sample surface. The acquired spectra were normalized to the incident photon flux and calibrated using Au 4 *f* and C 1 *s* core level peaks. The Shirley-Sherwood method was used for subtraction of the secondary electron background.

### STM measurements

The scanning tunneling microscopy measurements were carried out at 300 K in an ultrahigh vacuum with a base pressure of 2 × 10^−10^ Torr, using a home-built STM with commercial RHK R9 controller. The STM images were acquired in the constant-current mode using electrochemically etched polycrystalline W tips. All samples were cleaved in an ultrahigh vacuum by using scotch tape.

### Calculations

The first-principles calculations were performed by using a plane-wave basis, projector augmented wave potentials^37^ as implemented in Vienna ab initio simulation package (VASP)^38^. The kinetic energy cutoff for the plane waves was 300 eV. The exchange-correlation interaction of electron was treated by the Perdew-Burke-Ernzerhof implementation of the generalized gradient approximation^39^ in conjunction with the on-site Coulomb repulsion (*U* = 7 eV) for Gd *f*-orbital^40^. To describe long-ranged vdW interaction, DFT-D3 was applied^41^. We employed repeated slab geometry including 20Å-thick vacuum. Monkhorst-pack mesh of k-points (2 × 2× 1) was used for sampling the two-dimensional Brillouin Zone^42^. For the energetics, spin-polarized calculation was carried out using a 4×4 surface supercell containing 3QL slab. The atomic positions were relaxed until Hellmann-Feynman forces are smaller than 0.01 eV/Å^−1^. For the STM image simulations and core-level shift calculations^43^, a 6 × 6 surface supercell with 1QL is used and the spin-orbit coupling is explicitly treated^44^. (For the configurations with interstitial defects, a 2QL slab was employed.) The constant-current STM images were simulated within Tersoff-Hamann approximation^45,46^.

### Magnetic and electrical properties measurements

The magnetic and transport properties were measured in a temperature range of 2 K to 300 K with the superconducting quantum interference device-vibrating sample magnetometer (SQUID-VSM) up to 7 T. The magnetization was measured at 2 K and as sweeping magnetic field parallel to the cleaved surface of single crystals. In the same configuration, the magnetic susceptibility was measured in a magnetic field of 1 T. All transport data were taken using a dc four-probe method with conducting Pt wires. Magnetoresistance and Hall curves were obtained from the perpendicular configuration of the magnetic field perpendicular to the cleaved surface of single crystals.

## Supplementary information

Supplementary information

## Data Availability

The datasets analysed during the current study are available from the corresponding author on reasonable request.
