# Supplementary material for: Observation of Restored Topological Surface States in Magnetically Doped Topological Insulator
Source: Sci Rep. 2019 Feb 4;9:1331. doi: 10.1038/s41598-018-37663-8 (PMC6362033; doi:10.1038/s41598-018-37663-8)
Supplement: Supplementary file 1 — Supplementary information [file 41598_2018_37663_MOESM1_ESM.pdf]

Supplementary Information for "Observation of Restored Topological Surface States in Magnetically Doped Topological Insulator"

Jinsu Kim[1], Eun-Ha Shin[2], Manoj K. Sharma[3,4], Kyuwook Ihm[4], Otgonbayar Dugerjav[5], Chanyong Hwang[5], Hwangho Lee[6,7], Kyung-Tae Ko[6,7], Jae-Hoon Park[6,7,8], Miyoung Kim[2], Hanchul Kim[2*], Myung-Hwa Jung[1*]

[1]Department of Physics, Sogang University, Seoul 04107, Korea.

[2]Department of Physics, Sookmyung Women's University, Seoul 04310, Korea.

[3]Department of Applied Physics, Amity Institute of Applied Sciences, Amity University, Noida 201303, India.

[4]Pohang Accelerator Laboratory, Pohang 37673, Korea.

[5]Center for Nanometrology, Korea Research Institute of Standards and Science, Daejeon 34113, Korea.

[6]Department of Physics, Pohang University of Science and Technology, Pohang 37673, Korea.

[7]Max Planck POSTECH Center for Complex Phase Materials, Pohang University of Science and Technology, Pohang 37673, Korea.

[8]Division of Advanced Materials Science, Pohang University of Science and Technology, Pohang 37673, Korea.

*Corresponding authors' email; mhjung@sogang.ac.kr and hanchul@sookmyung.ac.kr

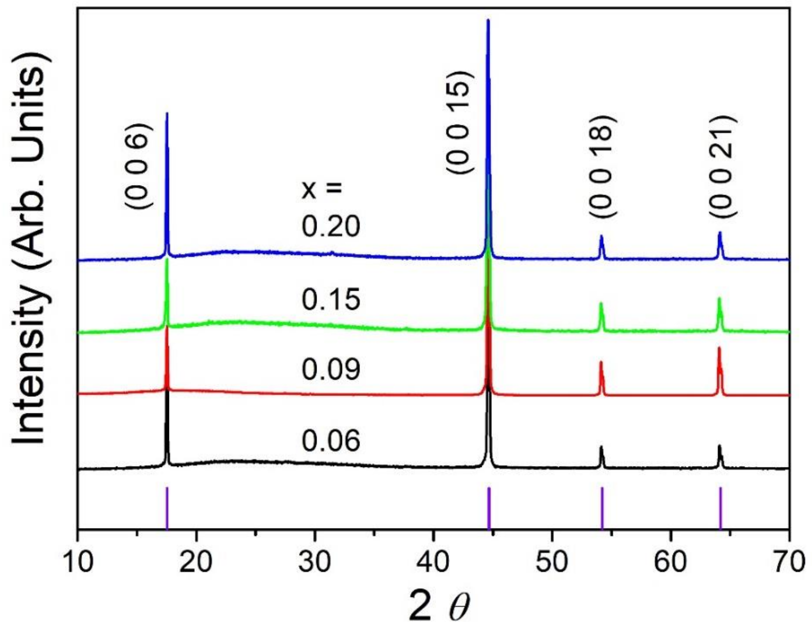

Figure S1. Single-crystal x-ray diffraction (XRD) patterns of $Gd_xBi_{2-x}Te_3$. The diffraction patterns possess the (003) family peaks without any impurity peaks, and the peak positions are in good agreement with the reference of Bi_2Te_3 (violet column, ICSD code. 15753). This result indicates that the $Gd_xBi_{2-x}Te_3$ crystallizes in a single crystal having the same structure as Bi_2Te_3. The lattice parameters are $a = 4.39 \pm 0.01$ Å and $c = 30.48 \pm 0.04$ Å, which are not affected by Gd substitution because of the small difference in ionic sizes between Bi and Gd.

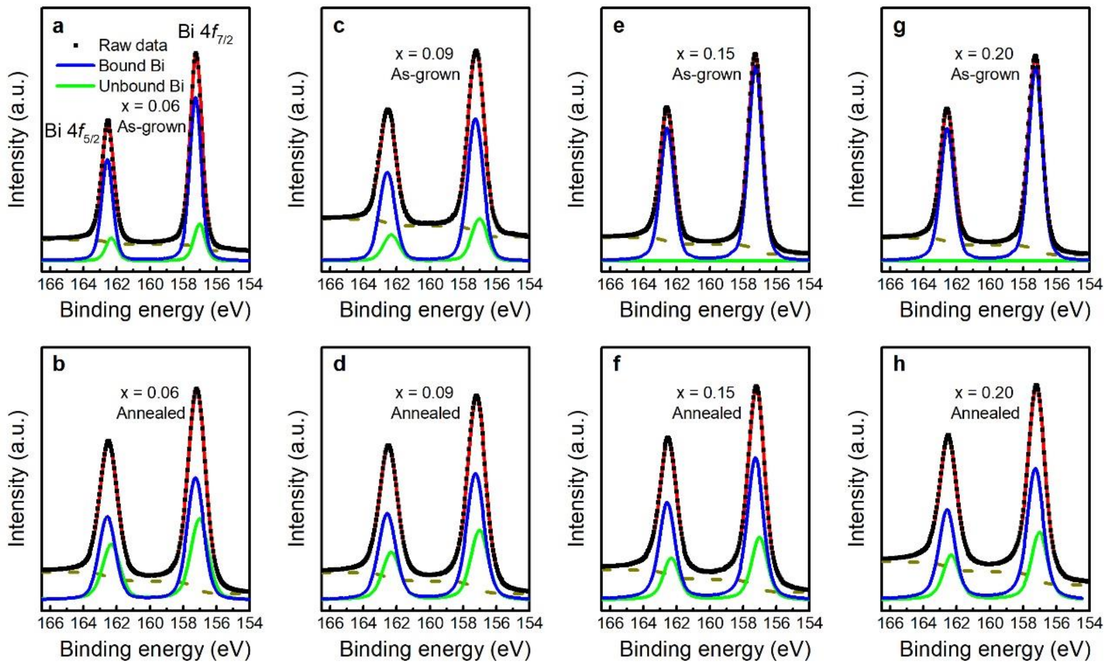

Figure S2. Bi 4*f* core-level x-ray photoelectron spectroscopy (XPS) spectra taken for as-grown and annealed $Gd_xBi_{2-x}Te_3$ single crystals. (a) to (d) ((e) to (h)) Bi 4*f* core-level XPS spectra of as-grown (annealed) samples for x = 0.06, 0.09, 0.15, and 0.20, respectively. Two main peaks around 157.0 and 162.3 eV correspond to the binding energy of Bi $4f_{7/2}$ and Bi $4f_{5/2}$, respectively. Each spectrum was fitted in the same way as described in the main text. The background signal is represented by a dashed line. The enhancement of unbound Bi components after annealing implies that the Bi ion breaks the bonds with the Te ions and is present on the surface as a metallic Bi state.

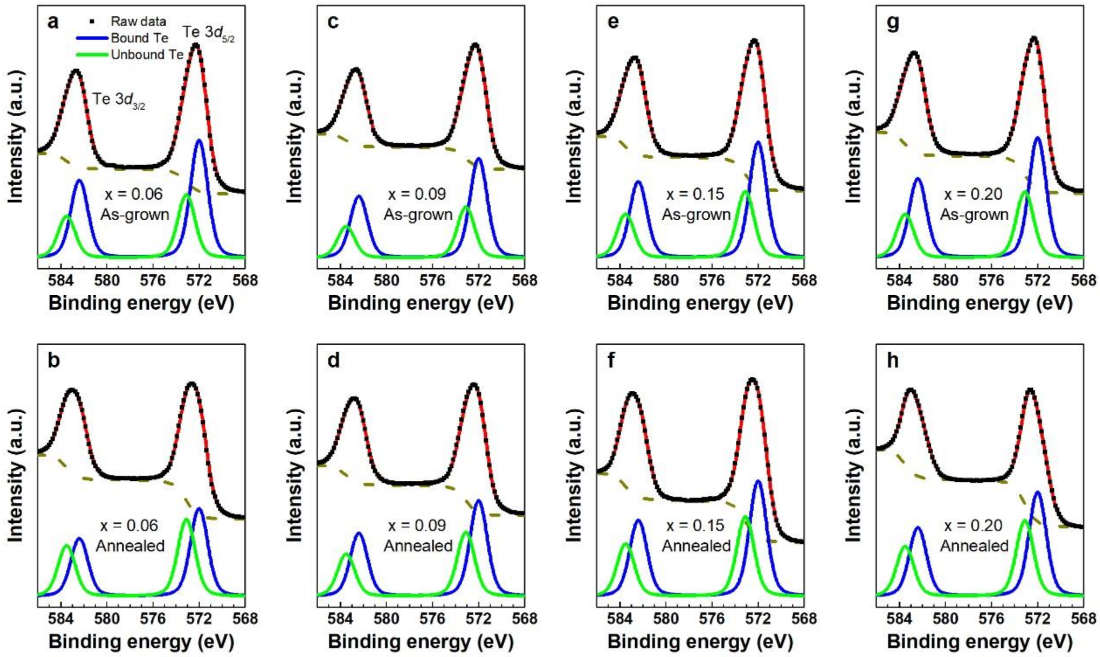

Figure S3. Te 3*d* core-level x-ray photoelectron spectroscopy (XPS) spectra taken for as-grown and annealed $Gd_xBi_{2-x}Te_3$ single crystals. (a) to (d) ((e) to (h)) Te 3*d* core-level XPS spectra of as-grown (annealed) samples for x = 0.06, 0.09, 0.15, and 0.20, respectively. Two main peaks around 572.4 and 582.8 eV correspond to the binding energy of Te $3d_{5/2}$ and Te $3d_{3/2}$, respectively. The spectra were fitted in the same way as the Bi 4*f* core-level analysis. The presence of unbound Te component in all the as-grown samples is due to the Te-rich crystal growth condition, which tends to generate the Te_{Bi}-type antisite defects. After annealing, deconvoluted results show that the unbound (bound) Te component is slightly increasing (decreasing), although the difference of area ratio for deconvoluted peaks is negligible between as-grown and annealed samples. Such small difference results from the volatile nature of Te atom during the annealing process. Note that oxygen-related peaks (i.e., Te-O bonds around 576.1 or 577.3 eV) are not detected.[1]

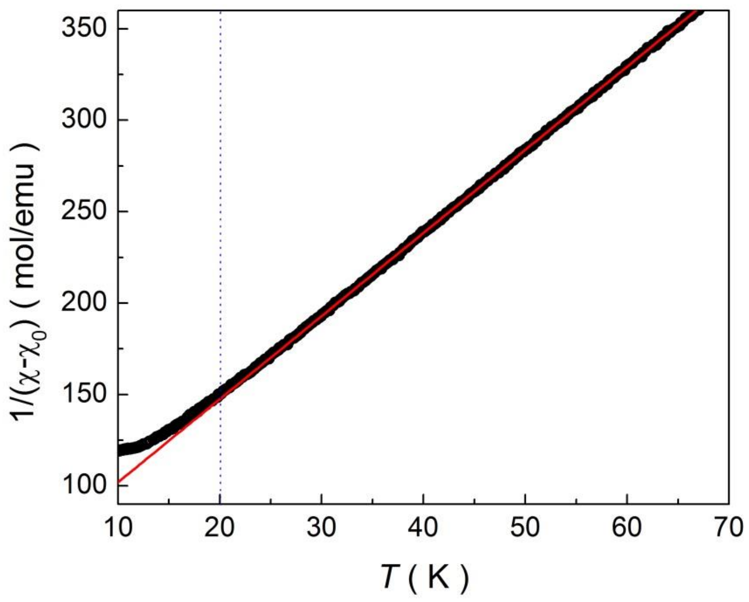

Figure S4. Magnetic susceptibility $\chi(T)$ measured at 1 T for as-grown sample as a function of temperature. The red line corresponds to the fitted curve with the Curie-Weiss law. The $\chi(T)$ data starts to deviate from the below 20 K, which is higher than T_N.

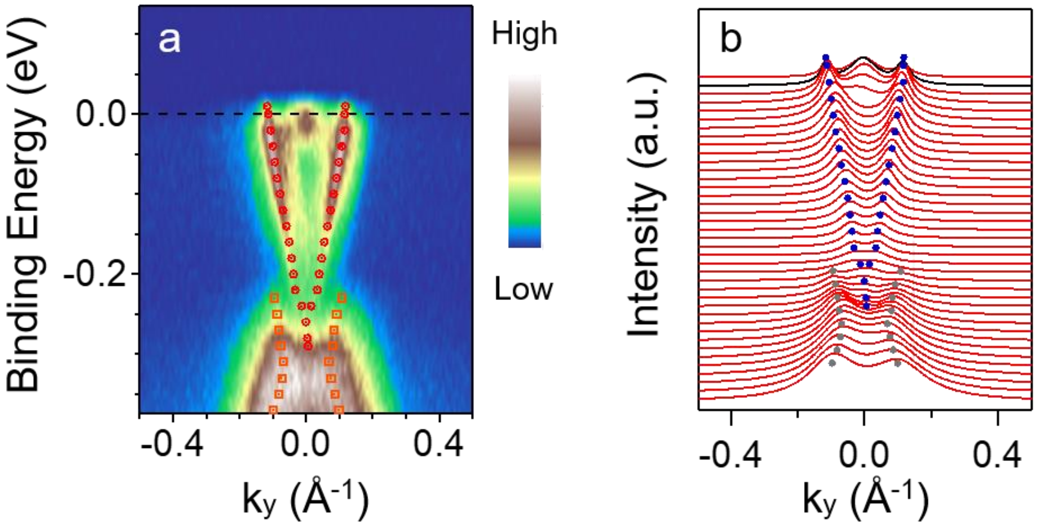

Figure S5. Band structure of c-annealed $Gd_{0.15}Bi_{1.85}Te_3$ single crystal measured at 20 K. Angle-resolved photoemission spectroscopy spectra (a) and the corresponding momentum distribution curves (b). The finite MDCs intensity near the Dirac point indicates that the surface band gap is closed after annealing. Also, the linear dispersion of the MDC peaks (blue dots) supports the gap closing, compared to the parabolic dispersion for as-grown sample in Fig. 2e with an energy gap opening.

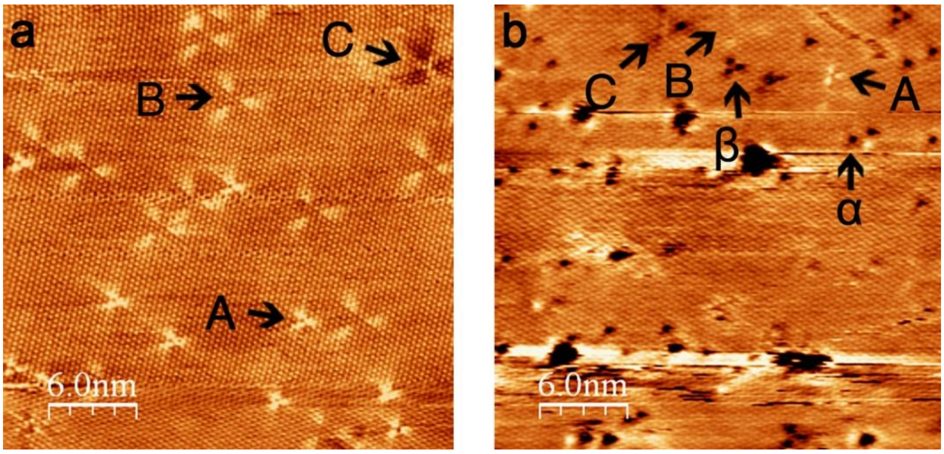

Figure S6. Empty-state STM image of pristine and Gd doped Bi_2Te_3 surface. (a) STM image of pristine (Te-rich) Bi_2Te_3 surface measured at V_S = +0.003 V. Native defects are denoted by a, b, and c. The defects a and b have clover-shaped protrusions, which are Te_{Bi} anti-site defects residing on the first and the second Bi layers (Te_{Bi1} and Te_{Bi2}), respectively. The defect c has clover-shaped depression, which is the Bi vacancy at the second Bi layer (V_{Bi2}). These defects were previously observed.[2,3] The bright features of a and b defects in the empty-state STM image indicate that they act as donors, while the dark c defect might act as an acceptor. (b) STM image of Gd-doped Bi_2Te_3 surface measured at V_S = +0.30 V. In addition to the defects A, B, and C observed in pristine Bi_2Te_3, the defects α and β are newly observed.

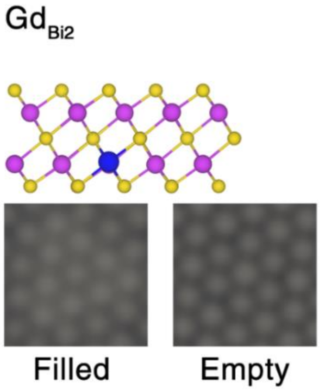

Figure S7. Simulated STM images of Gd_{Bi2} defect. The isolated Gd_{Bi2} defect is indistinguishable in the simulated STM image, in stark contrast to the case of substitutional transition metal in the Bi2 layer. [4,5] The filled- and empty-state images are simulated at V_S = -0.6 and +0.6 V, respectively.

Table S1. Formation energy of considered defect structures obtained by using a 4×4 supercell with a 3QL slab. The numbers in parentheses are obtained by using a 6×6 supercell with an 1QL slab (2QL for Gd_{Bi2}-Bi_I and Bi_I). The substitutional Gd at Bi sites (Gd_{Bi1} and Gd_{Bi2}) are the most stable configurations. The next stable structure is the Gd_{Bi2}-Bi_I pair.

Defects	Formation energy (eV)	
	**Te-rich condition**	**Bi-rich condition**
Gd_{ad}[top]	7.30 (7.30)	7.30 (7.30)
Gd_{ad}[fcc]	4.94 (4.94)	4.94 (4.94)
Gd_{ad}[hcp]	4.84 (4.41)	4.84 (4.41)
Gd_I	3.51 (3.06)	3.51 (3.06)
Gd_{Bi2}-Bi_I	3.33 (3.03)	3.33 (3.03)
Gd_{Bi1}	0.52 (0.01)	1.10 (0.59)
Gd_{Bi2}	0.52 (0.01)	1.11 (0.59)
Gd_{Bi1}-Bi_{Te1}	5.43 (7.64)	5.04 (6.09)
Gd_{Te1}	5.49 (-)	5.10 (-)
Bi_{Te1}	1.63 (1.96)	0.66 (0.99)
Bi_{Te3}	1.42 (1.96)	0.45 (0.99)
Bi_{ad}	2.07 (2.66)	1.49 (2.07)
Bi_I	3.12 (2.89)	2.54 (2.31)
V_{Bi1}	2.13 (2.19)	2.71 (2.77)
V_{Bi2}	2.04 (2.19)	2.63 (2.77)

We performed the first-principles calculation to examine stable Gd-related defects in Bi_2Te_3 surface. Single Gd atom can be adsorbed on the surface (Gd_{ad}), intercalated in the van der Waals (vdW) gap (Gd_I), and substituted for Bi (Gd_{Bi}) and Te (Gd_{Te}) atoms. There are three distinct adsorption sites; on-top of Te atom (top), on-top of Bi atom (hcp), and hollow (fcc)

sites. Likewise, Gd atom can occupy these three sites within the vdW gap. Supplementary Table S1 shows the formation energy (E_f) of all configurations calculated in this work.

The formation energy (E_f) was calculated by using the formula, $E_f = E[total] - E[Bi_2Te_3] - \sum \Delta n_i \cdot \mu_i$, where $E[total]$ is the total energy of the supercell containing defects and $E[Bi_2Te_3]$ is the total energy of the pristine Bi_2Te_3 surface. Δn_i is the change of the number of atoms (i = Gd, Bi, and Te) in the supercell. μ_i is the chemical potential of Gd, Bi, and Te. In the thermodynamic equilibrium for the stable growth of Bi_2Te_3, the chemical potentials of Bi and Te are not independent, $2\mu_{Bi} + 3\mu_{Te} = \Delta H_f(Bi_2Te_3)$, where ΔH is the formation enthalpy. We have $\mu_{Bi} = \mu_{Bi}^{bulk}$ in Bi-rich condition and $\mu_{Te} = \mu_{Te}^{bulk}$ in Te-rich condition. The possible range of chemical potentials are obtained, as follows. $\frac{1}{3}\Delta H_f(Bi_2Te_3) + \mu_{Te}^{bulk} \leq \mu_{Te} \leq \mu_{Te}^{bulk}$, $\frac{1}{2}\Delta H_f(Bi_2Te_3) + \mu_{Bi}^{bulk} \leq \mu_{Bi} \leq \mu_{Bi}^{bulk}$, and $\mu_{Gd} \leq \mu_{Gd}^{bulk}$. To avoid forming a Gd-Te compound, the Gd chemical potential is restricted: $\mu_{Gd} + 3\mu_{Te} \leq \Delta H_f(GdTe_3)$. The upper bound of μ_{Gd}, μ_{Gd}^{max}, depends on the crystal growth condition: μ_{Gd}^{max}(Te-rich condition) $<$ μ_{Gd}^{max}(Bi-rich condition) $<$ μ_{Gd}^{bulk}. The lower bound of μ_{Gd} is taken to be negative infinity. Since the Gd chemical potential is permitted under Bi- and Te-rich condition, μ_{Gd}^{max}=μ_{Gd}^{max}(Te-rich condition).

Supplementary Information References

1. Bahl, M. K., Watson, R. L. & Irgolic, K. J. X-ray photoemission studies of tellurium and some of its compounds. *J. Chem. Phys.* **66,** 5526-5535 (1977).

2. Jiang, Y. *et al.* Fermi-level tuning of epitaxial Sb_2Te_3 thin films on graphene by regulating intrinsic defects and substrate transfer doping. *Phys. Rev. Lett.* **108,** 066809 (2012).

3. Bathon, T. *et al*. Experimental realization of a topological p-n junction by intrinsic defect grading. *Adv. Mater*. **28,** 2193-2188 (2016).

4. Song, C.-L. *et al.* Gating the charge state of single Fe dopants in the topological insulator Bi_2Se_3 with a scanning tunneling microscope. *Phys. Rev. B* **86,** 045441 (2012).

5. Mann, C. *et al.* Observation of Coulomb repulsion between Cu intercalants in $Cu_xBi_2Se_3$. *Phys. Rev. B* **89,** 155312 (2014).
